# Inputs to quality: supervision, management, and community involvement in health facilities in Egypt in 2004

**DOI:** 10.1186/1472-6963-11-282

**Published:** 2011-10-20

**Authors:** Emily J Cherlin, Adel A Allam, Erika L Linnander, Rex Wong, Essam El-Toukhy, Heather Sipsma, Harlan M Krumholz, Leslie A Curry, Elizabeth H Bradley

**Affiliations:** 1 Section of Health Policy and Administration, School of Public Health, New Haven, CT, USA; 2 Al Azhar University and National Bank of Egypt, Cairo, Egypt; 3 Ministry of Health and Population, Egypt; 4 Robert Wood Johnson Clinical Scholars Program, Department of Medicine, Yale University School of Medicine, New Haven, Connecticut, USA; 5 Section of Cardiovascular Medicine, Department of Medicine, Yale University School of Medicine, New Haven, Connecticut, USA; 6 Center for Outcomes Research and Evaluation, Yale New-Haven Hospital, New Haven, Connecticut, USA

## Abstract

**Background:**

As low- and middle-income countries experience economic development, ensuring quality of health care delivery is a central component of health reform. Nevertheless, health reforms in low- and middle-income countries have focused more on access to services rather than the quality of these services, and reporting on quality has been limited. In the present study, we sought to examine the prevalence and regional variation in key management practices in Egyptian health facilities within three domains: supervision of the facility from the Ministry of Health and Population (MOHP), managerial processes, and patient and community involvement in care.

**Methods:**

We conducted a cross-sectional analysis of data from 559 facilities surveyed with the Egyptian Service Provision Assessment (ESPA) survey in 2004, the most recent such survey in Egypt. We registered on the Measure Demographic and Health Survey (DHS) website http://legacy.measuredhs.com/login.cfm to gain access to the survey data. From the ESPA sampled 559 MOHP facilities, we excluded a total of 79 facilities because they did not offer facility-based 24-hour care or have at least one physician working in the facility, resulting in a final sample of 480 facilities. The final sample included 76 general service hospitals, 307 rural health units, and 97 maternal and child health and urban health units (MCH/urban units). We used standard frequency analyses to describe facility characteristics and tested the statistical significance of regional differences using chi-square statistics.

**Results:**

Nearly all facilities reported having external supervision within the 6 months preceding the interview. In contrast, key facility-level managerial processes, such as having routine and documented management meetings and applying quality assurance approaches, were uncommon. Involvement of communities and patients was also reported in a minority of facilities. Hospitals and health units located in Urban Egypt compared with more rural parts of Egypt were significantly more likely to have management committees that met at least monthly, to keep official records of the meetings, and to have an approach for reviewing quality assurance activities.

**Conclusions:**

Although the data precede the recent reform efforts of the MOHP, they provide a baseline against which future progress can be measured. Targeted efforts to improve facility-level management are critical to supporting quality improvement initiatives directed at improving the quality of health care throughout the country.

## Background

Efforts to improve the quality of health care delivery have been central to health care reforms in the US and Europe during the last 30 years [1-3]; however, health reforms in low- and middle-income countries have more often focused on access to key services rather than the quality of these services. Nevertheless, as low- and middle-income countries experience economic development and increase national budgets for health care, ensuring quality of the health care delivery is a central component of health reform efforts.

Egypt is a particularly interesting country in which to examine health reform as it is a country in an epidemiologic transition [4], as is typical of countries developing from lower- to middle-income countries. Such an epidemiologic transition involves declining prevalence of infectious diseases and acceleration of chronic disease, such as cardiovascular disease, obesity, and cancer. In 2004, the percent of years of life lost in Egypt due to communicable disease was 31% versus 61% from non-communicable diseases [5]. Furthermore, as one of the only countries on target for achieving the Millennium Development Goals [6], Egypt and its health system are nonetheless under increasing pressure with the growing burden of chronic disease, emerging wealth among consumers demanding higher quality services, and persistent regional disparities in health [7]. In light of these pressures, the Ministry of Health and Population (MOHP) has implemented several health reforms including piloting social health insurance [8], establishing national health accounts and workforce projections for health planning [7], and developing accreditation systems for non-governmental facilities [9]. Additionally, the MOHP has engaged in targeted leadership and management training in rural health units of Upper Egypt, [10] and despite the recent political events, has initiated and sustained a Leadership Academy to build management capacity in hospitals. To date, national data have not been reported regarding the prevalence of key management practices than enable high quality care; such data are critical to ongoing evaluation of health reform efforts.

Accordingly, we sought to examine key management practices in Egyptian health facilities, using the Egyptian Service Provision Assessment (ESPA) 2004 survey [11]. We examined practices in three domains: supervision of the facility from the MOHP, managerial processes, and patient and community involvement in quality assurance. We also examined regional differences in these practices. Although the data precede the recent reform efforts of the MOHP, they provide a baseline against which future progress can be measured. Evidence from this study can be useful in evaluating ongoing efforts to improve health facility quality in Egypt.

## Methods

### Setting

The Egyptian health care system includes both public governmental and private health care providers. The MOHP is considered the principal provider with more than 5,000 facilities and more than 120,000 hospital beds. Private hospitals represent an additional 1,300 hospitals with about 26,000 beds [12]. Most services provided are free of charge or substantially subsidized and are generally managed through the Egypt's Governorate system. The Health Insurance Organization (HIO) system currently insures less than half of Egyptian citizens [13] although its expansion is planned to insure all citizens; private insurance does exist but is limited in scope currently [4].

### Sample

We conducted a cross-sectional analysis of data from the ESPA 2004 survey [11]. We registered on the Measurement Demographic and Health Survey (DHS) website http://legacy.measuredhs.com/login.cfm to gain access to the survey data. The ESPA 2004 survey, conducted jointly by the MOHP and El-Zanety Associates, was administered in a sample of 559 MOHP health facilities selected through systematic sampling of all government-owned MOHP facilities, stratified by Governorate. Private facilities were not included in the ESPA survey. From the ESPA sampled 559 MOHP facilities, we excluded a total of 79 facilities because they did not offer facility-based 24-hour care or did not have at least one physician working in the facility, yielding in a final sample of 480 facilities for analysis. The final sample of 480 facilities included 76 general service hospitals, 307 rural health units, and 97 maternal and child health and urban health units (MCH/urban units), representing approximately 10% of these types of facilities in Egypt. Rural health units are facilities in the non-urban settings that provide primary care, immunizations, and laboratory services to residents in their catchment areas; urban health units include primary care facilities in the urban settings as well as those clinics that focus on maternal and child health.

### Measures

We examined outcomes in 3 domains: 1) external supervision by the MOHP of the health facility, 2) managerial processes within the facility, and 3) community and patient involvement. The first domain was assessed with a binary variable for whether the facility had received supervision visit in the last 6 months, as well as the reported activities included in the visit (e.g., checked registers, discussed problems, observed staff). The second domain was assessed with binary variables indicating whether the facility: 1) had a management committee that met at least monthly, 2) kept official records from management committee meetings, 3) had a system for monitoring the quality of care, 4) kept written documentation of quality assurance activities (e.g., used supervisory checklists, conducted mortality reviews, audited medical records, documented the existence of quality assurance teams, or documented quality improvement programs), or 5) had an approach for reviewing quality assurance activities (i.e., management committees, quality assurance committees, or region or district management teams). The third domain was assessed with binary variables indicating whether the facility: 1) had joint meetings of facility managers and community members, 2) had a procedure for collecting and reporting patient feedback, and 3) had instituted changes based on patient feedback within the previous 3 months. We also used data on the facility location, which we categorized into the three main regions of Egypt: Urban Egypt (i.e., Cairo, Alexandria, Port Said and Suez), Lower Egypt (i.e., 9 governorates in the Nile Delta), and Upper Egypt (i.e., 8 predominantly rural governorates in the Nile Valley) [14].

### Statistical Analysis

We used standard frequency analyses to describe facility characteristics and the prevalence of external supervision, managerial processes, and community and patient involvement in quality assurance. We examined the associations between each outcome measure and the facility location, and we tested the statistical significance of these associations using chi-square statistics. We conducted separate analyses for general service hospitals, for rural health units, and for MCH/urban health units. All analyses were performed using SAS Version 9.1.3 (Carey, NC, 2002-2003) and were weighted according to ESPA 2004 recommendations to account for unequal sampling probabilities.

## Results

### General Service Hospitals

Nearly 60% of general service hospitals (n = 76) were located in Lower Egypt, about one third were located in Upper Egypt, and only 7% were located in Urban Egypt (Table 1). External supervision within the previous 6 months was reported by the vast majority of hospitals across all regions. In contrast, managerial processes were far less prevalent, particularly in Upper and Lower Egypt. In Lower Egypt, less than half of hospitals had regular management meetings. Less than 10% performed quality assurance activities, which was significantly fewer than in Urban Egypt or Upper Egypt. Across all regions, hospitals were particularly low in measures of community and patient involvement.

**Table 1:** Organizational characteristics of General Hospitals (n = 76)*

Characteristic	All Regions (n = 76)	Urban Egypt^a ^ (n = 5)	Lower Egypt^b ^ (n = 45)	Upper Egypt^c ^ (n = 26)
	**# (%)**	**# (%)**	**# (%)**	**# (%)**

**External Supervision**				

Facility received external supervision	75 (98.7)	5 (100.0)	45 (100.0)	25 (96.2)
within the previous 6 months	69 (90.8)	4 (80.0)	42 (93.3)	23 (88.5)
Checked registers/books	65 (85.5)	5 (100.0)	39 (86.7)	21 (80.8)
Discussed problems	48 (63.2)	4 (80.0)	26 (57.8)	18 (69.2)
Discussed policy issues	47 (61.8)	4 (80.0)	24 (53.3)^bc^	19 (73.1)^bc^
Discussed technology problems	18 (23.7)	2 (40.0)	6 (13.3)^bc^	10 (38.5)^bc^
held an official staff meeting	56 (73.7)	5 (100.0)	33 (73.3)	18 (75.8)
Observed individual staff at work	57 (75.0)	3 (60.0)	35 (77.8)	19 (73.1)
Recorded observations				

**Managerial Processes**				
Facility has a management committee that meets every month or more	38 (50.0)	5 (100.0)^ab; ac^	19 (42.2)	14 (53.8)
Facility keeps official records from the management committee meetings	29 (38.2)	4 (80.0)^ab^	14 (31.1)^ab^	11 (42.3)
Facility has a system for monitoring the quality of care that is delivered	15 (19.7)	4 (80.0)^ab; ac^	4 (8.9)^ab; bc^	7 (26.9)^ac; bc^
Facility keeps written documentation of quality assurance activities	14 (18.4)	4 (80.0)^ab; ac^	4 (8.9)^ab^	6 (23.1)^ac^
Facility has an approach for reviewing quality assurance activities	14 (18.4)	4 (80.0)^ab; ac^	4 (8.9)^ab^	6 (23.1)^ac^

**Community/patient involvement**				
Facility holds meetings with *both *facility managers and community members	39 (51.3)	4 (80.0)	20 (44.4)	15 (57.7)
Facility has a procedure to collect and report patient feedback	3 (3.9)	1 (20.0)	2 (4.4)	0 (0.0)
Facility has instituted changes within the previous 3 months, based on patient feedback	5 (6.6)	1 (20.0)	2 (4.4)	2 (8.0)

**^ab ^**Urban facilities different from Lower Egypt facilities (p < 0.05)

**^ac ^**Urban facilities different from Upper Egypt facilities (p < 0.05)

**^bc ^**Lower Egypt facilities different from Upper Egypt facilities (p < 0.05)

^* ^Percent calculated using non-missing as denominator [missing number range (0 - 1)]

### Rural Health Units

Among rural health units (n = 307), receipt of external supervision within the previous 6 months was reported by nearly 99% of facilities (Table 2). Less than 25% of facilities had any of the managerial processes present in any of the regions. Furthermore, community and patient involvement was particularly low, reported in fewer than 5% of rural health units. Significant differences were apparent between Upper and Lower Egypt rural health units, with Upper Egypt facilities being significantly less likely to use systems for quality assurance and to hold joint management and community meetings.

**Table 2:** Organizational characteristics of Rural Health Units

Characteristic	All Regions (n = 307)*	Lower Egypt^b ^ (n = 171)	Upper Egypt^c ^ (n = 136)
	**# (%)**	**# (%)**	**# (%)**

**External Supervision**			
Facility received external supervision	304 (99.0)	171 (100.0)	133 (97.8)
within the previous 6 months	295 (96.1)	167 (97.7)	128 (94.1)
Checked registers/books	239 (77.9)	119 (69.6)^bc^	120 (88.2)^bc^
Discussed problems	194 (63.2)	94 (55.0)^bc^	100 (73.5)^bc^
Discussed policy issues	199 (64.8)	104 (60.8)^bc^	95 (79.9)^bc^
Discussed technology problems	121 (39.4)	65 (38.0)	56 (41.2)
held an official staff meeting	212 (69.1)	109 (63.7)^bc^	103 (75.7)^bc^
Observed individual staff at work	266 (86.6)	155 (90.6)	111 (81.6)
Recorded observations			

**Managerial processes**			
Facility has a management committee that meets every month or more	79 (26.0)	41 (24.3)	38 (28.1)
Facility keeps official records from the management committee meetings	24 (7.9)	17 (10.1)	7 (5.2)
Facility has a system for monitoring the quality of care that is delivered	45 (14.9)	32 (19.3)^bc^	13 (9.6)^bc^
Facility keeps written documentation of quality assurance activities	37 (12.1)	29 (17.0)^bc^	8 (5.9)^bc^
Facility has an approach for reviewing quality assurance activities	37 (12.1)	27 (15.8)^bc^	10 (7.4)^bc^

**Community/patient involvement**			
Facility holds meetings with *both *facility managers and community members	51 (16.9)	36 (21.6)^bc^	15 (11.1)^bc^
Facility has a procedure to collect and report patient feedback	10 (3.3)	8 (4.7)	2 (1.5)
Facility has instituted changes within the previous 3 months, based on patient feedback	12 (3.9)	7 (4.2)	5 (3.7)

**^bc ^**Lower Egypt facilities different from Upper Egypt facilities (p < 0.05)

^* ^Percent calculated using non-missing as denominator [missing number range (0 - 6)]

### MCH/Urban Health Units

Among the MCH/urban health units (n = 97), nearly all facilities reported receiving external supervision from the MOHP within the previous 6 months (Table 3). Significantly fewer facilities in Upper or Lower Egypt than in Urban Egypt had routine and documented management meetings, although the use of quality assurance systems, rare overall, did not differ significantly by region. Furthermore, the use of community and patient involvement was low among the MCH/urban health units, although health units in Urban Egypt were significantly more likely than health units in both Lower and Upper Egypt to hold joint management and community member meetings and to have a procedure for collecting and reporting on patient feedback.

**Table 3:** Organizational characteristics of MCH/Urban Health Units

Characteristic	All Regions (n = 97)*	Urban Egypt^a ^ (n = 19)	Lower Egypt^b ^ (n = 40)	Upper Egypt^c ^ (n = 38)
	**# (%)**	**# (%)**	**# (%)**	**# (%)**

**External Supervision**				
Facility received external supervision	95 (97.9)	19 (100.0)	40 (100.0)	36 (94.7)
within the previous 6 months	95 (97.9)	19 (100.0)	40 (100.0)	36 (94.7)
Checked registers/books	87 (89.7)	19 (100.0)	36 (90.0)	32 (84.2)
Discussed problems	62 (63.9)	14 (73.7)	23 (57.8)	25 (65.8)
Discussed policy issues	80 (82.5)	18 (94.7)	31 (77.5)	31 (81.6)
Discussed technology problems	42 (43.3)	11 (57.9)^ac^	22 (55.0)^bc^	9 (23.7)^bc^
held an official staff meeting	86 (88.7)	19 (100.0)	36 (90.0)	31 (81.6)
Observed individual staff at work	82 (84.5)	17 (89.5)	32 (80.0)	33 (86.8)
Recorded observations				

**Managerial Processes**				
Facility has a management committee that meets every month or more	40 (41.2)	16 (84.2)^ab; ac^	11 (27.5)^ab^	13 (34.2)^ac^
Facility keeps official records from the management committee meetings	20 (20.6)	10 (52.6)^ab; ac^	5 (12.5)^ab^	5 (13.2)^ac^
Facility has a system for monitoring the quality of care that is delivered	23 (27.1)	3 (16.7)	9 (31.0)	11 (28.9)
Facility keeps written documentation of quality assurance activities	20 (20.6)	3 (15.8)	9 (22.5)	8 (21.1)
Facility has an approach for reviewing quality assurance activities	23 (23.7)	3 (15.8)	9 (22.5)	11 (28.9)

**Community/patient involvement**				
Facility holds meetings with *both *facility managers and community members	41 (42.3)	16 (84.2)^ab; ac^	13 (32.5)^ab^	12 (31.6)^ac^
Facility has a procedure to collect and report patient feedback	2 (2.1)	2 (10.5)^ab; ac^	0 (0.0)^ab^	0 (0.0)^ac^
Facility has instituted changes within the previous 3 months, based on patient feedback	3 (3.1)	1 (5.3)	0 (0.0)	2 (5.3)

**^ab ^**Urban facilities different from Lower Egypt facilities (p < 0.05)

**^ac ^**Urban facilities different from Upper Egypt facilities (p < 0.05)

**^bc ^**Lower Egypt facilities different from Upper Egypt facilities (p < 0.05)

^* ^Percent calculated using non-missing as denominator [missing number range (0 - 11)]

## Discussion

Our findings highlight that, as of 2004, most health facilities had external supervision within the last 6 months; however, key facility-level management practices were lacking across all types of government-owned health facilities (hospital, rural health units, and MCH/urban health units) in Egypt. During this time before the current MOHP, less than half of the health facilities reported having a regular management committee meeting at least monthly. Less than one third of facilities reported having any quality assurance systems. Such management systems have been shown to be associated with better quality care [15,16] and can be particularly meaningful in translating new policies for health care improvement into practice. Although external supervision from the MOHP can be helpful, without internal management meetings, it is unlikely that facilities will adapt well to the increasing health services demands of a growing economy and aging population. These results reflect substantial needs for management capacity building in government health care facilities in Egypt, which will become more marked with the current political instability of the country.

We also found marked variation in health facility management practices by region in Egypt. Many managerial processes were significantly more prevalent in health facilities in Urban Egypt compared with Lower or Upper Egypt. Among hospitals, disparities between urban and rural facilities were most apparent in the implementation of quality assurance systems. Among health units, disparities between urban and rural facilities were most apparent in the involvement of community and patients in care, which was generally uncommon. The findings underscore the need for targeted efforts in management capacity building outside the urban Egypt. Furthermore, community and patient involvement, which was limited in Egypt, may be an untapped opportunity to gain valuable customer feedback for facility-based quality improvement efforts.

Additionally, the most commonly observed managerial practice was the use of management meetings. However, record keeping and documentation of related data was rare throughout both urban and rural regions of the country. This finding is consistent with experience in low- and middle-income countries [17], and it highlights a fundamental hurdle in improving quality of care delivery in such settings. Investment in data monitoring and analysis capability within the health sector will be important to effect positive changes in the quality of care for the Egyptian population.

Our results should be interpreted in light of their limitations. First, although the ESPA data provide a large national sample of government-owned facilities, the depth of measures available is limited. For instance, although management meetings may have been held, we cannot assess the quality or impact of these meetings. This limitation highlights the opportunity to develop a more comprehensive, locally-appropriate, systematic tool to evaluate management systems across health facilities in Egypt to better understand variation in management practices and guide investment in focused improvement. For instance, such a tool could include measures of management effectiveness such as staff competencies, waiting times for services, stock outs in pharmacy, human resources practices, and other key performance indicators reflecting management systems. Second, the data were collected in 2004, and the recent efforts of the MOHP and other stakeholders may have changed the situation in targeted facilities. Nevertheless, these findings will contribute to the evaluation of ongoing efforts by the MOHP to improve management capacity of health facilities and quality of care across Egypt. Third, the data are self-reported and may overestimate the prevalence of quality assurance or other processes, although reported frequencies were already markedly low in most management practices. Additionally, we lacked comparison data for other countries at a similar level of development. Last, we did not use patient-level data and therefore were unable to evaluate the quality of clinical care in these facilities. Although additional measures pertinent to patient care are essential to a comprehensive understanding of facility quality, our findings are an important first step in describing the functioning of management practices within government-owned health facilities.

## Conclusions

Recent health reform efforts in Egypt have generally targeted important systems-level levers, such as financing and workforce planning, to improve accessibility to of health services, especially in rural areas. Although these reforms are important, this study highlights a gap in the facility-level managerial processes that underpin high quality healthcare service delivery. These data will provide a useful baseline for ongoing evaluation of current efforts by the MOHP and its Leadership Academy. The Egyptian experience highlights the need to build management capacity as an integral part of health reforms. With the recent political events in Egypt, efforts to reform and strengthen the health care system continue to be important, as provision of high quality health care can be a central in developing a strong and peaceful citizenry. Although political and civil unrest can distract from ongoing programs to improve social services, having strong health services remains critical during times of peace and unrest. This is particularly important in middle income countries, as robust health systems will be required to effectively respond to rapid epidemiologic and economic transitions.

## Competing interests

The authors declare that they have no competing interests.

## Authors' contributions

Authors responsible for conception and design of study included EC, EL, HS and EB. EC contributed to acquisition of the data. Authors responsible for the analysis and interpretation of the data included EC, AA, EE, EL, HS, and EB. Drafting and critical revision of the manuscript included EC, AA, EL, RW, EE, HS, HK, LC, and EB. Last, Final approval of the manuscript included EC, AA, EL, RW, EE, HS, HK, LC, and EB.

## Pre-publication history

The pre-publication history for this paper can be accessed here:

http://www.biomedcentral.com/1472-6963/11/282/prepub
